# Estimation of Infiltration Parameters and the Irrigation Coefficients with the Surface Irrigation Advance Distance

**DOI:** 10.1371/journal.pone.0101274

**Published:** 2014-07-25

**Authors:** Zhou Beibei, Wang Quanjiu, Tan Shuai

**Affiliations:** 1 State Key Laboratory Base of Eco-Hydraulic Engineering, Institute of Water Resources and Hydro-electric Engineering, Xi'an University of Technology, Xi'an, Shaanxi, China; 2 State Key Laboratory of Soil Erosion and Dryland Farming on the Loess Plateau, Institute of Soil and Water Conservation, Chinese Academy of Sciences & Ministry of Water Resources, Northwest A & F University, Yangling, Shaanxi, China; Zhejiang University, China

## Abstract

A theory based on Manning roughness equation, Philip equation and water balance equation was developed which only employed the advance distance in the calculation of the infiltration parameters and irrigation coefficients in both the border irrigation and the surge irrigation. The improved procedure was validated with both the border irrigation and surge irrigation experiments. The main results are shown as follows. Infiltration parameters of the Philip equation could be calculated accurately only using water advance distance in the irrigation process comparing to the experimental data. With the calculated parameters and the water balance equation, the irrigation coefficients were also estimated. The water advance velocity should be measured at about 0.5 m to 1.0 m far from the water advance in the experimental corn fields.

## Introduction

Border irrigation and surge flow irrigation method have been applied in arid and semi-arid regions to conserve water and to increase water productivity since 1987 in China [1]. Both of these irrigation methods have the advantages of achieving more uniform irrigation, saving water resources by speeding up irrigation water advance and reducing deep percolation [2]–[3], which was wide spread in China. Infiltration properties of the soil and irrigation coefficients are among the most important factors in the design and management of irrigation systems, but it is often difficult to be obtained because of the spatial and temporal variability of soil properties [4]–[5]. Due to the importance of irrigation parameters in surface irrigation system design and evaluation, numerous investigations are being conducted to measure or predict the surface irrigation events such as advance, storage, recession and subsurface [6]–[10]. Even though direct measurements are technically preferable, the parameters obtained may not be applicable at field scale [11]. As a result, indirect evaluation of field parameters by numerical inversion of the governing equations has become an effective alternative to directive methods for the estimation of surface irrigation parameters. Three are several methods to estimate the surface irrigation parameters, such as one-point method, two-point method, as well as advance-repercussion method. The one-point method and two-point method could only estimate the surface irrigation parameters in a flat, homogenous field [12]–[13]; moreover, the advance-repercussion method could obtain the surface irrigation parameter only with the mean water depth [14]or the water storage in the field after the irrigation [15]. The review of literature suggests that even though various methods are available for the estimation of surface irrigation parameters, the needed information, such as irrigation advance, recession, and irrigation water depth data for the parameters, are very hard to be obtained in the field with an accuracy value except of the advance time and water advance distance.

Thus, the objective of this study was to develop a simple method based on Manning roughness equation, Philip equation and the volume balance equation to predicate the border irrigation and surge flow irrigation parameters and infiltration parameters only with the water advance trajectory which is easy to be measured accurately in a surface irrigation experiment.

## Model Development

### Border irrigation

The unit width flux and water flow velocity for irrigation could be described by the manning equation as:
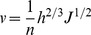
(1)

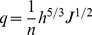
(2)


where, v is the water advance velocity, L/T; q is the unit width flux, V/T; h is the depth of flow, H; J is the slope degree; n is the manning roughness coefficient.

In order to analyze the irrigation advance process, we assume that the depth of irrigation water among the distance be described with an exponential function as follows:
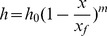
(3)


Where h_0_ is the depth of surface water at the inlet, H; x is the distance to the inlet, L; x_f_ is the surface water advance distance, L; m is a shape coefficient of the surface water profile.

Furthermore, in order to determine the water advance velocity, we assume that the water advance velocity at x_f_-a may represent the velocity of water advance velocity, and the corresponding water depth will be:
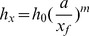
(4)


Correspondingly, the water advance velocity at x_f_-a is:
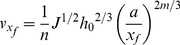
(5)


There is a functional relationship of the advance distance with the advance velocity:
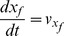
(6)


Connecting Eq. (5) with Eq. (6):
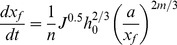
(7)


Integrating Eq. (7):
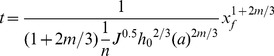
(8)


Let D = 1+2m/3, and then Eq. (8) becomes:
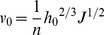
(9)


and Eq. (8) could be written as: 
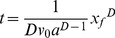
(10)


Eq. (10) describe the relationship between the surface water advance distance and the time.

Soil infiltration characteristics are usually expressed in a time-dependant infiltration equation. One of the most common equations is the Philip equation:
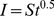
(11)


Where I is the cumulative infiltration, L; S is the sortivity, L/T^1/2^.

The water starts infiltrating into the subsurface soil at a point along the border strip only after the irrigation front reaches that point. Denoting t_x_ as the net infiltration time at ‘x’ point,

And then,
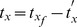
(12)


Where, t_xf_, x_t′_ are the time that the irrigation front reaches to x_f_ point and x point, respectively.

According to eq (11), the infiltration at the x_f_ point could be described as:
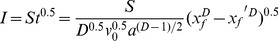
(13)


As a result, the total infiltration amount (F), from the inlet to the distance x_f_ is:
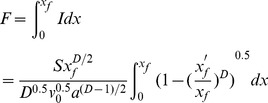
(14)


Eq (18) could be simplified as follows:
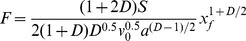
(15)


When water reaches the point, x_f_, the mount of surface water storage is:
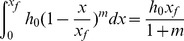
(16)


Therefore, the water balance equation could be written as:
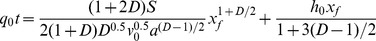
(17)


### Surge irrigation

Similar to the theory improvement in Border irrigation, the theory was improved in the same way. During the surge irrigation process, when each surge was conducted, the irrigation time was measured as t(i),

The unit width flux and water flow velocity for each irrigation surge could be described by the manning equation as:
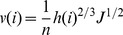
(18)

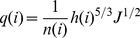
(19)


where, v(i) is the water advance velocity for each surge, L/T; q(i) is the unit width flux for each surge, V/T; h(i) is the depth of flow in each surge, H; J is the slope degree; n(i) is the manning roughness coefficient for each surge.

In order to analyze the irrigation advance process, we assume that the depth of irrigation water among the distance be described with an exponential function as follows:
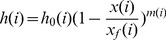
(20)


Where h_0_ (i) is the depth of surface water at the inlet in each surge, H; x(i) is the distance to the inlet in each surge, L; x_f_(i) is the surface water advance distance in each surge, L; m(i) is a shape coefficient of the surface water profile in each surge.

Furthermore, in order to determine the water advance velocity, we assume that the water advance velocity at x_f(i)_-a(i) may represent the velocity of water advance, and the corresponding water depth will be:
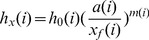
(21)


Correspondingly, the water advance velocity 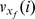 at x_f_(i)-a(i) is:
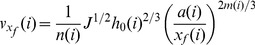
(22)


There is a functional relationship of the advance distance with the advance velocity:
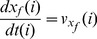
(23)


Connecting Eq. (22) with Eq. (23):
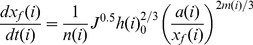
(24)


Integrating Eq.(24):
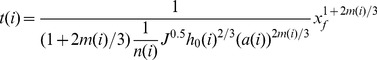
(25)


Let D(i) = 1+2m(i)/3, and then Eq.(8) becomes:
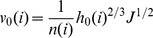
(26)


and Eq.(8) could be written as: 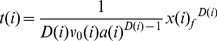
(27)


Eq.(27) describe the relationship between the surface water advance distance and the time.

Soil infiltration characteristics are usually expressed in a time-dependant infiltration equation. One of the most common equations is the Philip equation shown as follows,
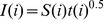
(28)


Where I(i) is the cumulative infiltration in each surge, L; S is the sortivity in each surge, L/T^1/2^.

The water starts infiltrating into the subsurface soil at a point along the border strip only after the irrigation front reaches that point. Denoting t_x_(i) as the net infiltration time at ‘x’ point,

And then,
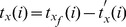
(29)


Where, t_xf_, x_t′_ are the time that the irrigation front reaches to x_f_ point and x point in each surge, respectively.

According to eq (29), the infiltration at the x_f_ point in each surge could be described as:
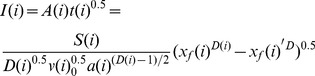
(30)


As a result, the total infiltration amount (F), from the inlet to the distance x_f_ is:
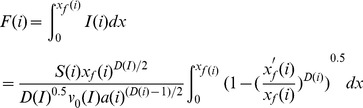
(31)


Eq (31) could be simplified as follows:
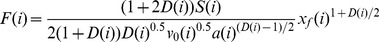
(32)


When water reaches the point, x_f_(i), the mount of surface water storage in each surge could be described as,
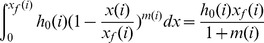
(33)


Therefore, the water balance equation could be written as:
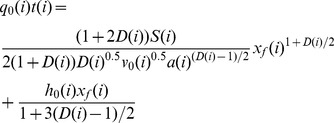
(34)


## Materials and Methods

The field experiments were conducted in Bayinguoleng Mongolia Autonomous prefecture Bazhou irrigation station which was one of the field stations of our Scientific team (Prof. Wang Quanjiu was response for experimental fields in this manuscript and can be contacted in the future). And I declare that my manuscript does not matter the any ethic issue.

Bayinguoleng Mongolia Autonomous prefecture Bazhou irrigation station was classified as a warm-temperature arid zone with continental climate. The field experiments were carried out on 3 bays (4*200 m) in a corn field. Corn is an important crop here which requires an average of 6 irrigations; however, the irrigation time may be increased to 7–9 irrigations in drier climate.

The measured values of the physical properties of the soil are given in Table 1, including the soil bulk density and percentage of sand, silt and clay, at the depths of 0–10 cm, 10–20 cm, 20–30 cm, 30–40 cm, 40–50 cm, 50–60 cm. The soil at the experimental site is classified as sandy soil.

**Table 1:** Soil characteristics of the experiment field.

Depth (cm)	Density (g/cm^3^)	clay (%) <0.002 mm	silt (%) 0.002∼0.02 mm	sand (%) 0.02∼2 mm
0–10	1.53	5.3	22.7	72
10–20	1.51	6.6	26.9	66.5
20–30	1.48	5.9	24.8	69.3
30–40	1.49	5.9	24.5	69.6
40–50	1.45	5.6	23.3	71.1
50–60	1.59	2.4	8.8	88.8
Average	1.51	5.3	21.8	72.9

In both of the border irrigation and surge irrigation experiments, irrigation water were pumped onto the bay through a weir plate which could measure the water flow rate, and then was shut off when the advance front reached a distance of 200 m. The flow rate was about 3.94 L/m·s. Border irrigation advance time was recorded that when the advance front arrived at a known distance. The soil water contents of the soil profile before and after the border irrigation were measured and then be used to verify our improved theories. In our surge irrigation, there were three cycles of on-time and off-time periods. Water advances to the end of the field only during the last surge. During each surge, water advances and infiltrates in the same way as that in a continuous irrigation. In each cycle, the water contents in the soil profiles before and after the surge irrigation were also measured, as well as the infiltration rates measured with a two-double ring method in order to certify the proposed theory. Both the border irrigations and surge irrigations were conducted 3 times.

## Results and Discussions

### Border irrigation

**Figure 1:**
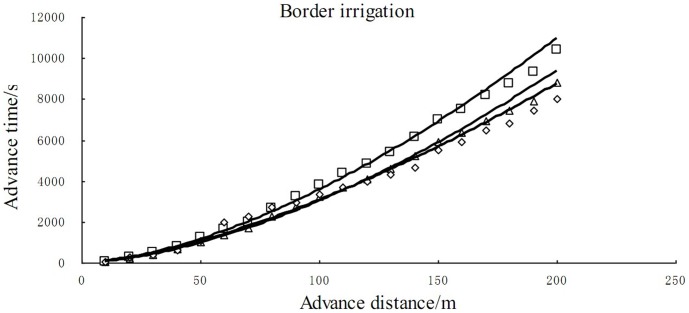
Comparison of the measured and fitted water advance process.

Trials of the models have been carried out using three experimental data sets for discussions. In order to understand the water advance phase under the border irrigation, results from experimental site were shown in Fig.1. From Fig.1 it can be easily found that the water advanced distance increased with the time increased. In order to evaluate the theory, the water advancing distance changing with the time was fitted well by a power function and the parameters were shown in Table.2.

Based on the measured water advanced curves, the parameters of V_0_×a∧(1-D) and D were easily obtained, thus the parameters of S, a, h_0_ could be obtained with eq(10) and eq(17) according to the volume balance equation. Once the parameter of h_0_ is known, the roughness coefficient could be calculated with equation (1) or equation (2) shown in Table 2.

**Table 2:** Fitted parameters from the measured water advance process.

replications	V_0_×a∧(1-D)	D/m/s^1/2^	R^2^
1	2.006	1.619	0.998
2	2.239	1.511	0.986
3	1.636	1.634	0.998

**Figure 2:**
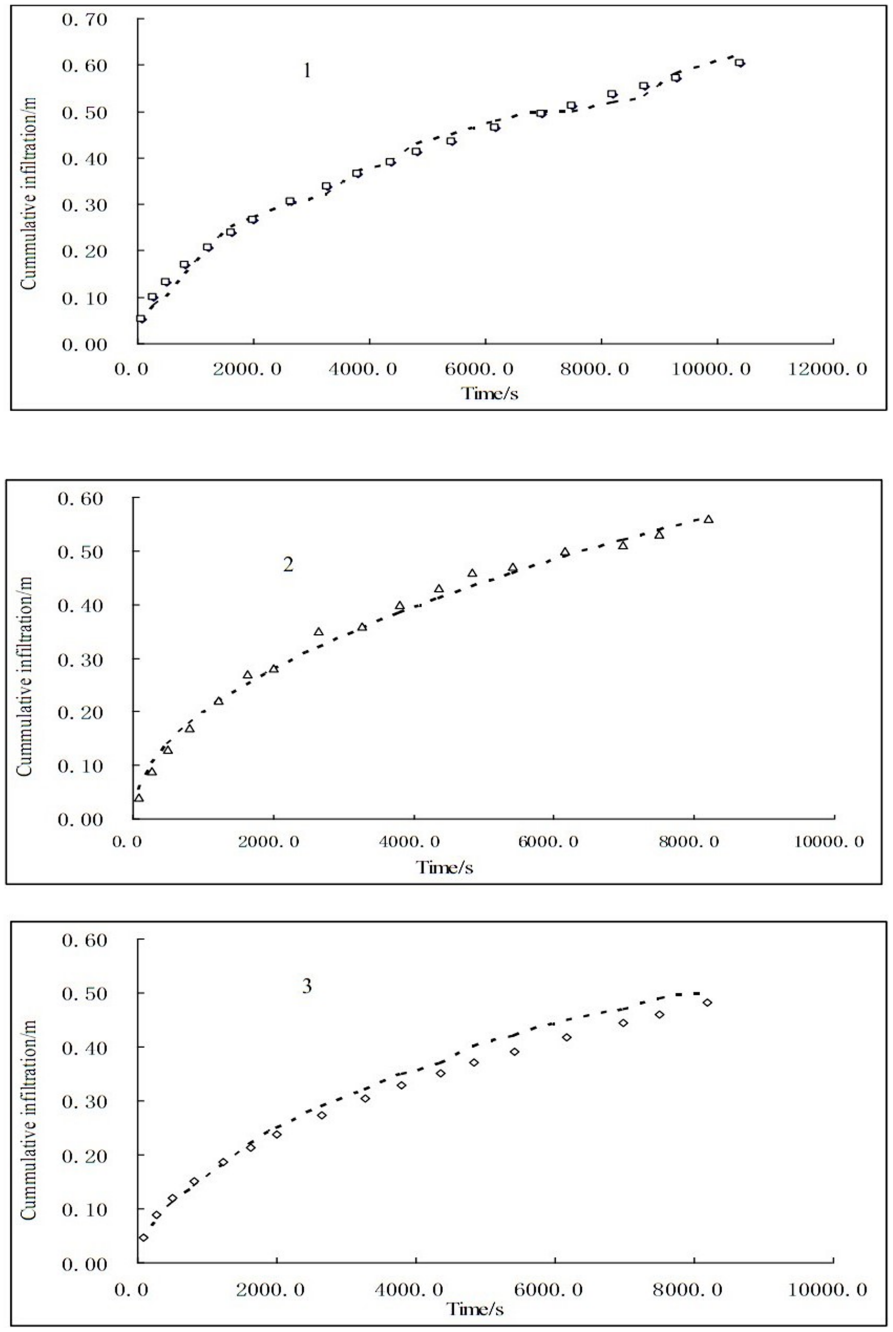
comparing the measured infiltration curve of the border irrigation with the simulated infiltration curve.

From Table 3, we can found that the average value of parameter α may represent the water advance velocity before the water advance 1.007 m that may represent the true water advance velocity. The irrigation water depth was about 3.9 cm which was also credible in the irrigation event. Then with the parameters above, the cumulative infiltration changing with the time could be estimated as follows shown in Fig.2. From Fig.2, it could be found that the new developed theory could estimate the infiltration process very well.

**Table 3:** Fitted parameters with the improved theory.

replications	α/m	A m/s∧^0.5^	h_0_/cm	n
1	1.003	0.048	3.9	0.036
2	1.200	0.073	4.1	0,038
3	0.818	0.049	3.7	0.035
average	1.007	0.057	3.9	0.036

### Surge irrigation

Similar to Border irrigation process, in order to certify our improved theory about the surge irrigation, the surge irrigation experiments were also conducted in the Corn field. The experimental field was 2.5 m in width, 200 m in length. Each experiment includes three cycles of on-time and off-time periods. The time for the first surge irrigation starts at 0 min, the second surge irrigation starts at 120 min, and the last surge irrigation starts at 240 min. Water advances to the end of the field only during the last surge. During each surge, water advances and infiltrates in the same way as that in a continuous irrigation.

**Figure 3:**
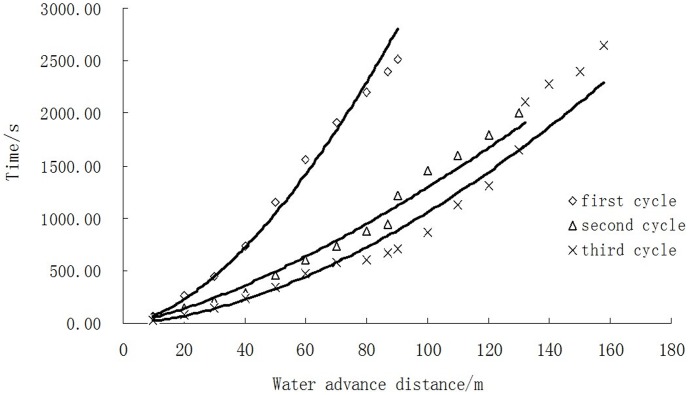
Water advance process in three cycles of Surge irrigation.

In order to describe the surge irrigation process clearly, the advance distance in a certain time was shown in Fig.3 and the process was fitted by a power function. Then the needed parameters could be obtained from the fitted results shown in Table 3.

According to the equation(19) to (34), the parameters could be calculated as follows in Table 4.

**Table 4:** Mean fitted parameters of the water advance process in all the three cycles.

	D m/s1/2	V_0_×a∧(1-D)	R^2^
First cycle	1.6771	1.4791	0.9931
Second cycle	1.4926	1.5053	0.9694
Third cycle	1.6984	0.4236	0.9876

**Figure 4:**
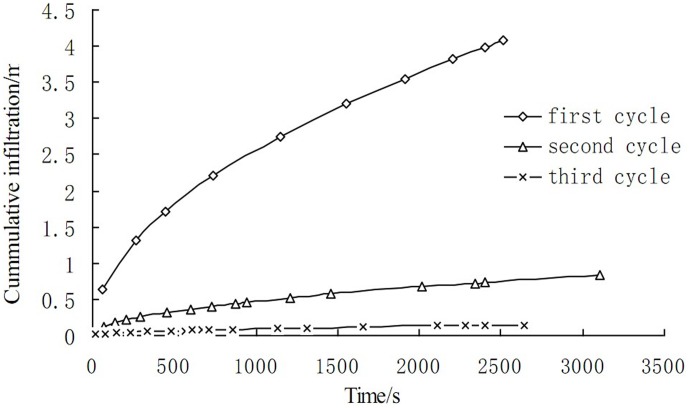
Estimated infiltration curve of the border irrigation.

Table 5 shows the mean fitted parameters in three irrigation cycles. The infiltration processes of all the three surge irrigations were also estimated shown in Fig.4. From Fig.4 it could be seen that the cumulative infiltration in a certain time decreased greatly after the 1st irrigation and 2nd irrigation. Since the soil of the wetting surface becomes nearly saturated after the first surge, the enlarged water film around the soil particles decreases the surface tension of the soil particle, leading to a reduced aggregation, in addition to the mechanical effect of water flow scouring, surface soil disintegrated, soil structure is destroyed and the particle distribution of the surface is changed, A sealing layer is formed on the field surface after the suspended soil particles deposit as the ponded surface water dries up. As a result, water advance velocity increased while the roughness coefficient and the irrigation water depth decreased from the first cycle to third cycle shown similar results to Wang [1] and Fei [13]. (Fig.4). The development of this sealing layer reduces the roughness and intake rate of the wetting soil surface, creating a smooth surface to the next surge to advance quickly towards the end of the field, thus the roughness coefficient decreased after the first cycle.

**Table 5:** Mean fitted parameters in three irrigation cycles.

	First cycle	Second cycle	Third cycle
A/m/s∧^0.5^	0.081	0.029	0.0018
H_0_/cm	3.7	2.0	2.0
N	0.020	0.007	0.007
v_0_/m/s	0.35	0.639	0.639
a/m	1.23	0.587	0.586

## Conclusions

The results of this study suggest that infiltration parameters and irrigation coefficient could be calculated more accurately only from the advance data both in the border irrigation and the surge irrigation. In our corn filed, the water advance velocity would be measured after the water advance about 0.5 m to 1.0 m. Furthermore, the advance data were very easy to be obtained and were much more reliability comparing to other measured data which were important characteristic in this study.
